# The Mucosally-Adherent Rectal Microbiota Contains Features Unique to Alcohol-Related Cirrhosis

**DOI:** 10.1080/19490976.2021.1987781

**Published:** 2021-11-07

**Authors:** Ting-Chin David Shen, Scott G. Daniel, Shivali Patel, Emily Kaplan, Lillian Phung, Kaylin Lemelle-Thomas, Lillian Chau, Lindsay Herman, Calvin Trisolini, Aimee Stonelake, Emily Toal, Vandana Khungar, Kyle Bittinger, K. Rajender Reddy, Gary D. Wu

**Affiliations:** a Division of Gastroenterology and Hepatology, Department of Medicine, University of Pennsylvania Perelman School of Medicine, Philadelphia, Pennsylvania, USA; b Division of Gastroenterology, Hepatology, and Nutrition, Children’s Hospital of Philadelphia, Philadelphia, Pennsylvania, USA

**Keywords:** Alcohol-related cirrhosis, gut microbiota, mucosal microbiome, rectal swab

## Abstract

Most studies examining correlations between the gut microbiota and disease states focus on fecal samples due to ease of collection, yet there are distinct differences when compared to samples collected from the colonic mucosa. Although fecal microbiota has been reported to be altered in cirrhosis, correlation with mucosal microbiota characterized via rectal swab has not been previously described in this patient population. We conducted a cross-sectional analysis using 39 stool and 39 rectal swabs from adult patients with cirrhosis of different etiologies and performed shotgun metagenomic sequencing. Bacterial growth studies were performed with *Escherichia coli*. Two asaccharolytic bacterial taxa, *Finegoldia magna* and *Porphyromonas asaccharolytica*, were increased in rectal swabs relative to stool (FDR < 0.01). Genomic analysis of the microbiome revealed 58 genes and 16 pathways that differed between stool and rectal swabs (FDR < 0.05), where rectal swabs were enriched for pathways associated with protein synthesis and cellular proliferation but decreased in carbohydrate metabolism. Although no features in the fecal microbiome differentiated cirrhosis etiologies, the mucosal microbiome revealed decreased abundances of *E. coli* and Enterobacteriaceae in alcohol-related cirrhosis relative to non-alcohol related cirrhosis (FDR < 0.05). *In vitro* bacterial culture studies showed that physiological concentrations of ethanol and its oxidative metabolites inhibited *E. coli* growth in a pH- and concentration-dependent manner. Characterization of the mucosally associated gut microbiome via rectal swab revealed findings consistent with amino acid/nitrogen abundance versus carbohydrate limitation in the mucosal microenvironment as well as unique features of alcohol-related cirrhosis possibly consistent with the influence of host-derived metabolites on the composition of mucosally adherent microbiota.

## Introduction

It is increasingly recognized that the gut microbiota plays important roles in health and disease through its interactions with host immunity and metabolism.^1,2^ Of particular interest is the relationship between the gut microbiota and the liver. The liver receives approximately 70% of its vascular supply from the portal vein, which provides a portal of entry for gastrointestinal contents including diet, alcohol, and xenobiotics to enter the systemic circulation after undergoing first-pass hepatic metabolism.^3^ At the same time, the gut microbiota and its metabolites can translocate across the intestinal epithelium to enter the portal vein, whereby the liver functions to mitigate or enhance their systemic effects upon the host.^4,5^ Conversely, hepatic metabolites such bile acids, urea, and acetaldehyde enter the gastrointestinal tract via the biliary tract and/or diffusion from systemic circulation into the colon where they may have an effect on the gut microbiota.^6,7^ The bidirectional communication and exchange between the gut and the liver form the so-called gut–liver axis and has led to increasing interests in understanding and exploring the role of the gut microbiota in liver diseases such as cirrhosis.

Dysbiosis, or alterations in gut microbiota composition and function in association with disease states, has been widely characterized in cirrhosis through both animal and human studies. Although not all studies have identified the same taxonomic alterations, consistent findings in cirrhosis have revealed increased Proteobacteria and Fusobacteria phyla, Enterobacteriaceae and Streptococcaceae families, and decreased commensal bacterial taxa such as Lachnospiraceae, Ruminococcaceae, and Clostridiaceae.^8,9^ Additionally, there may be small intestinal bacterial overgrowth as well as an invasion of oral/buccal microbiota into the lower intestinal tract, likely secondary to altered bile acid production, intestinal dysmotility, compromised mucosal immune defenses (e.g. antimicrobial peptides and IgA), and use of medications that reduce gastric acid production.^6,10^ Importantly, intestinal permeability is increased in cirrhosis,^11^ leading to heightened risks for the translocation of microbes and/or microbial products such as endotoxin to enter the host. This not only leads to infectious complications in cirrhosis, but also trigger Toll-like receptors on hepatic stellate and Kupffer cells, worsening hepatic inflammation and fibrosis.^3^ Furthermore, the host immune system has been shown to be dysfunctional in cirrhosis.^12^ The combination of these factors predisposes patients with cirrhosis to increased risks of infectious and inflammatory complications, culminating in systemic conditions such as bacteremia, hepatic encephalopathy (HE), spontaneous bacterial peritonitis (SBP), and acute-on-chronic liver failure (ACLF).

A better characterization of the gut microbiota and its physiological interactions with the host through the gut–liver axis holds promise in improving the care of patients with liver disease. Within the gastrointestinal tract, longitudinal differences exist in the gut microbiota due to changes in nutrient availabilities, pH, oxygen, and bile acids along the length of the gut. At the same time, radial differences may exist between the luminal and mucosally adherent gut microbiota due to additional factors such as proximity to the gastrointestinal mucosal immune system and oxygen gradient secondary to diffusion from the intestinal epithelium.^13^ Mucosally adherent gut microbiota generally comprise more aerotolerant, facultative anaerobes such as Enterobacteriaceae that include many bacterial taxa associated with pathogenicity and dysbiosis.^13^ Given increased intestinal permeability in the setting of cirrhosis and likelihood for translocation,^14^ understanding the mucosally adherent gut microbiota may be particularly important in this patient population.

Stool is widely used in gut microbiome studies due to ease of collection but represents primarily the colonic luminal gut microbiota and not the mucosally adherent microbiota. Mucosal biopsies obtained via flexible sigmoidoscopy or colonoscopy capture mucosally adherent microbiota, but are time-consuming, expensive, and invasive to perform. Additionally, mucosal biopsies are generally obtained after a bowel prep with a purgative such as polyethylene glycol, which has been shown to alter the gut microbiome as well as mucosal integrity.^15,16^ In comparison with stool and mucosal biopsies, rectal swabs have been under-utilized in gut microbiome studies but are readily accessible, relatively noninvasive, and simple to perform and store for subsequent processing. Gut microbiome characterization via rectal swab has been shown to be highly reproducible and closely resemble characterization via mucosal biopsies when not visibly soiled with stool.^17,18^ It has been utilized to characterize the gut microbiota in healthy adults and infants as well as in diseased states including inflammatory bowel diseases, acute pancreatitis, and critical illness.^17–21^ Although rectal swabs have been used in cirrhosis to detect specific drug-resistant pathogens using traditional culture techniques,^22,23^ to our knowledge, no study has utilized rectal swab to comprehensively survey the gut microbiome via sequencing-based technologies in cirrhosis. In this study, we set out to compare luminal and mucosally adherent gut microbiota in cirrhosis via stool and rectal swab, respectively, using shotgun metagenomic sequencing, with the notion that observed differences might be reflective of the different microenvironments in which they reside.

## Results

### Characterization of study population

We conducted a cross-sectional analysis of 39 stool and 39 rectal swabs without visible stool contamination (including paired rectal swab-stool samples from 33 patients) using baseline samples from cirrhosis patients enrolled in the ACCLIMATE (Acute-on-Chronic Liver Failure with Gut Microbiota-Targeted Assessment and Treatment) study. ACCLIMATE is a longitudinal cohort study that investigates changes in the gut microbiome before *versus* after and with *versus* without the development of acute-on-chronic liver failure (ACLF) in patients with cirrhosis over time. All samples analyzed in this current study are baseline samples from stable outpatients with cirrhosis; no patient was diagnosed with ACLF, hospitalized for treatment of cirrhosis-related complications, or on intravenous antibiotics at the time of sample collection. Table 1 describes the patient characteristics by sample types. Overall, there were no significant differences in patient characteristics between rectal swab and stool samples in terms of age, gender, race, etiology of cirrhosis, complications of cirrhosis, severity of disease based on Child-Pugh Score (CPS) and Model for End-Stage Liver Disease (MELD) score, and use of oral antibiotics, lactulose, or proton pump inhibitors (PPI).

**Table 1.** Characteristics of the study cohort by sample type

	Stool (*n* = 39)	Rectal swab (*n* = 39)
Age (mean±SD)	58.1 ± 10.4	57.7 ± 10.7
Gender (% female)	38.5	43.6
Race (% Caucasian)	76.9	74.4
CPS (mean±SD)	7.90 ± 1.29	7.62 ± 1.2
MELD (mean±SD)	15.87 ± 6.31	14.74 ± 5.08
Cirrhosis etiology (%)		
EtOH	41	41
NASH	25.6	23.1
HCV	15.4	17.9
PBC/PSC	7.7	7.7
Granulomatous	0	2.6
Multifactorial	10.3	7.7
Cirrhosis complications (%)		
HE	61.5	64.1
Ascites	92.3	94.9
SBP	5.1	5.1
Varices	43.6	48.7
HCC	2.6	2.6
Medications (%)		
Any antibiotic	53.8	59.0
Rifaximin	41	48.7
Lactulose	51.3	53.8
PPI	41	46.2

*Abbreviations: CPS, Child-Pugh Score; MELD, Model for End-Stage Liver Disease; EtOH, ethanol; NASH, nonalcoholic steatohepatitis; HCV, hepatitis C virus; PBC, primary biliary cholangitis; PSC, primary sclerosing cholangitis; HE, hepatic encephalopathy; SBP, spontaneous bacterial peritonitis; HCC, hepatocellular carcinoma; PPI, proton pump inhibitor.*

### The fecal and mucosal microbiome are distinct in cirrhosis

**Figure 1.**
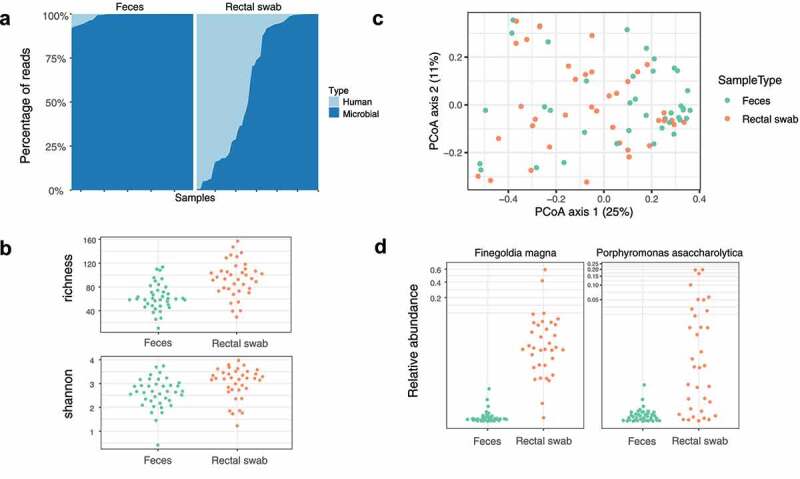
Shotgun metagenomic sequencing of fecal and rectal swab samples in cirrhosis. (a) Human vs. microbial DNA reads of fecal and rectal swab samples. (b) Alpha-diversity indices of fecal and rectal swab samples (richness *p* < .001, Shannon *p* = .0065). (c) Principal Coordinates Analysis of fecal and rectal swab samples via Bray-Curtis distances (*p* = .006, *R*^2^ = 0.04). (d) Relative abundances of *Finegoldia magna* (FDR 2.5 × 10^−24^) and *Porphyromonas asaccharolytica* (FDR 4.7 × 10^−9^) in fecal and rectal swab samples

We performed shotgun metagenomic sequencing in stool and rectal swabs to compare luminal and mucosally adherent microbiota, respectively. Quality control of DNA reads showed that rectal swab samples contained higher proportions of human DNA than fecal samples (Figure 1a), likely due to the abundance of the intestinal epithelium in mucus. Rectal swab samples exhibited greater α-diversity relative to fecal samples (Figure 1b; richness, *p* < .001; Shannon diversity, *p* = .0065). β-diversity was also different as measured by Bray–Curtis distances (Figure 1c; *p* = .006), although the effect size was small (R^2^ = 0.04). The relative abundances of two specific asaccharolytic bacterial taxa, *Finegoldia magna* (FDR 2.5 × 10^−24^) and *Porphyromonas asaccharalytica* (FDR 4.7 × 10^−9^), were significantly increased in rectal swab relative to stool (Figure 1d; Supplemental Figure S1).

*Shotgun metagenomic analysis reveals features consistent with amino acid/nitrogen abundance versus carbohydrate limitation in the rectal mucosa relative to lumen*

**Figure 2.**
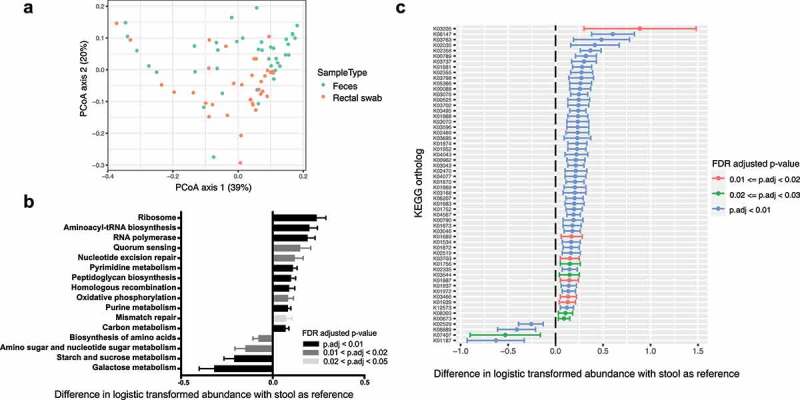
Gene and pathway abundance analysis of fecal and rectal swab samples in cirrhosis. (a) Principal Coordinates Analysis of gene abundances via Bray–Curtis distances in stool and rectal swabs (*p* = .003; *R*^2^ = 0.059). (b) Sixteen pathways based on KEGG with >1% abundance that differ significantly between stool and rectal swabs. Positive value indicates greater abundance in rectal swab, and negative value indicates greater abundance in stool. Shading signifies degree of statistical significance. (c) 58 genes based on KEGG with >0.1% abundance that differ significantly between stool and rectal swabs. Positive value indicates greater abundance in rectal swab, and negative value indicates greater abundance in stool. Color-coding signifies degree of statistical significance

β-diversity of gene abundances differed between rectal swab and stool as measured by Bray-Curtis distances (Figure 2a, FDR = 0.003, R^2^ = 0.059). Using linear models of logistic transformed abundances, we discovered 16 pathways (Figure 2b) with mean abundance >1% that differed significantly between rectal swabs and stool (FDR < 0.05). Of these 16 pathways, 12 are increased in rectal swabs relative to stool. In descending order, the three most increased pathways are ribosome, aminoacyl-tRNA biosynthesis, and RNA polymerase, indicating greater protein synthesis in the rectal mucosa relative to lumen. Of the four pathways that are decreased in rectal swabs, three pathways are involved in sugar and carbohydrate metabolism, and the fourth pathway relates to biosynthesis of amino acids.

Using linear models of logistic transformed abundances, we also found 58 genes with mean abundance >0.1% (Figure 2c) that differed significantly between rectal swabs and stool (FDR < 0.05). Of these 58 genes, 54 genes are increased in rectal swab relative to stool and encode various proteins involved in cellular proliferation and protein biosynthetic processes (Supplemental Table S1). The four genes that are decreased in rectal swab relative to stool encode three proteins involved in sugar/carbohydrate metabolism and one uncharacterized protein. Overall, along with increased abundances of the two asaccharolytic taxa found in the rectal swabs, these findings are consistent with carbohydrate limitation and/or amino acid/nitrogen abundance in the mucosal microenvironment compared to the lumen.

### Rectal swab, but not stool, reveals microbial signature unique to alcohol-related cirrhosis

**Figure 3.**
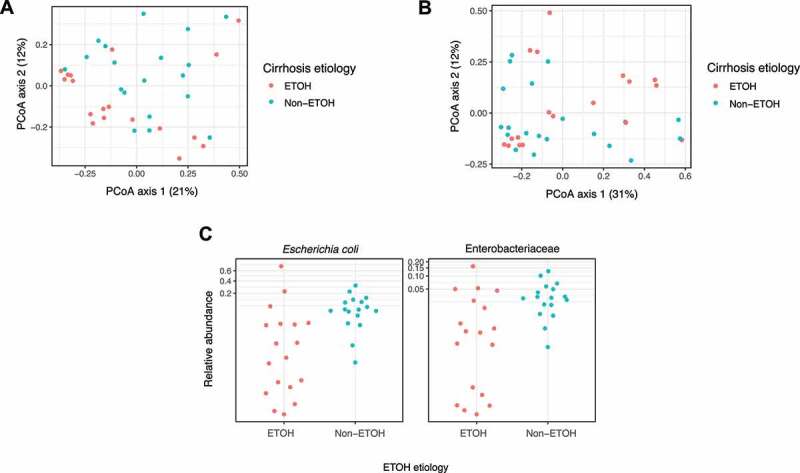
Beta-diversity of EtOH vs non-EtOH cirrhosis with linear model of taxonomic changes. (a) Principal Coordinates Analysis via Bray–Curtis distances of rectal swabs by EtOH vs. non-EtOH etiology of cirrhosis (*p* = .19; *R*^2^ = 0.03). (b) Principal Coordinates Analysis via Bray–Curtis distances of stool by EtOH vs. non-EtOH etiology of cirrhosis (*p* = .31; *R*^2^ = 0.03). (c) Relative abundances of *E. coli* and Enterobacteriaceae in rectal swabs of EtOH vs. non-EtOH cirrhosis (FDR < 0.05). Gray dotted line indicates separation of high and low *E. coli*/Enterobacteriaceae groups

Previous reports in the literature show conflicting results on whether the gut microbiome differs according to etiology of cirrhosis.^8,9,24^ We sought to determine whether luminal and/or mucosa-associated gut microbiota distinguished cirrhosis etiology in our patient cohort. β-diversity as measured by Bray-Curtis distances failed to reveal distinct clustering by cirrhosis etiology in either rectal swab or stool (Supplemental Figure S2). Since alcohol-related (EtOH) cirrhosis comprised the majority of our samples, we determined whether microbial compositions differed between EtOH and non-EtOH cirrhosis. β-diversity as measured by Bray–Curtis distances was not statistically different between EtOH and non-EtOH cirrhosis in either rectal swab or stool (Figure 3a,b). However, linear models revealed a taxonomic signature that distinguished EtOH from non-EtOH cirrhosis in the rectal swab, where relative abundances of *E. coli* and Enterobacteriaceae in EtOH cirrhosis were significantly lower than non-EtOH cirrhosis (FDR < 0.05; Figure 3c). This was not seen in the stool, and no significant differences in patient characteristics were found between EtOH cirrhosis and non-EtOH cirrhosis in terms of age, gender, race, complications of cirrhosis, disease severity as measured by CPS and/or MELD scores, and use of antibiotics, lactulose, or PPI. There were no differences in alpha-diversity or genes/pathways between EtOH and non-EtOH cirrhosis either in stool or rectal swab (Supplemental Figure S3).

Closer examination of rectal swabs from patients with EtOH cirrhosis revealed two clusters based on relative abundances of *E. coli* and Enterobacteriaceae: one cluster with relative abundances similar to those of subjects with non-EtOH cirrhosis (“high *E. coli*/Enterobacteriaceae group”), and another cluster with relative abundances close to zero (“low *E. coli*/Enterobacteriaceae group”) (Figure 3c). Patients in the low *E. coli*/Enterobacteriaceae group trended toward having more recent alcohol consumption than patients in the high *E. coli/*Enterobacteriaceae group (*p* = .11, Supplemental Figure S4a). Of the 18 patients with EtOH cirrhosis, 2 patients reported active alcohol consumption, and 16 patients reported last consuming alcohol ranging from 2 months to 12 years prior to sample collection. Information on the total duration, amount, and type/concentration of alcoholic beverages consumed was not collected in this study but can potentially affect *E. coli*/Enterobacteriaceae abundance. High and low *E. coli*/Enterobacteriaceae groups did not differ based on age, gender, complications of cirrhosis, disease severity as measured by CPS or MELD, and use of antibiotics or PPI. Interestingly, there was an association between *E. coli*/Enterobacteriaceae abundance and lactulose use, where eight of eleven subjects in the high *E. coli*/Enterobacteriaceae group were on lactulose, and only one of seven subjects in the low *E. coli*/Enterobacteriaceae group was on lactulose (*p* < .05). Additionally, there was an association with Caucasian ethnicity, as all eleven subjects in the high *E. coli/*Enterobateriaceae group were Caucasians, and only two out of seven subjects in the low *E. coli/*Enterobacteriaceae group were Caucasians (*p* < .01). There were no differences in Bray–Curtis distances of KEGG term abundances between high- and low *E. coli*/Enterobacteriaceae groups or with non-EtOH cirrhosis (Supplemental Figure S4b).

### In vitro studies reveal concentration- and pH-dependent inhibitory effects of ethanol and its oxidative metabolites on E. coli growth

**Figure 4.**
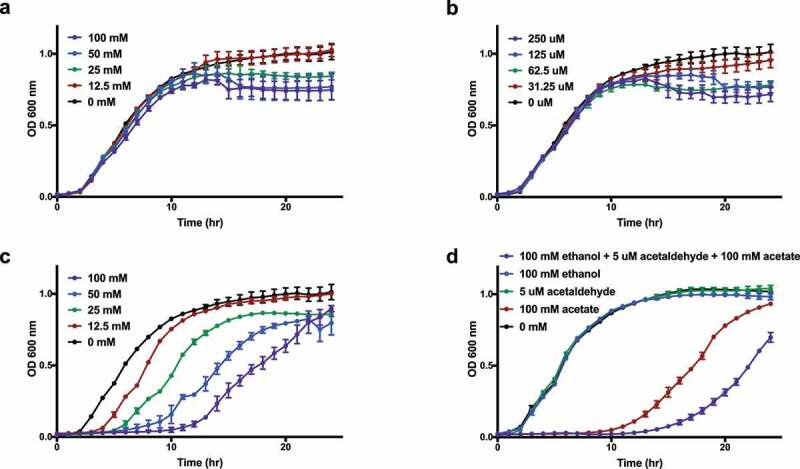
*E. coli* growth *in vitro* under physiological concentrations of ethanol after alcohol consumption and its oxidative metabolites at pH 6. Growth curve in LB (aerobic, 37°C) with (a) ethanol, (b) acetaldehyde, (c) acetate, and (d) ethanol, acetaldehyde, and acetate alone and in combination. Data expressed as mean ± SEM. *N* = 3 replicates per condition

Given that taxonomic differences between EtOH versus non-EtOH etiologies of cirrhosis were only seen in rectal swab and not stool, we hypothesized that it may be secondary to changes in the mucosal microenvironment due to diffusion of ethanol from systemic circulation into the colon at equivalent concentrations after alcohol intake,^25,26^ preferentially affecting mucosa-associated gut microbiota. To test this hypothesis, we incubated *E. coli* type strain ATCC 11775 *in vitro* in the presence of different physiologic concentrations of ethanol from alcohol consumption. Since ethanol can be oxidatively metabolized into acetaldehyde and acetate via the enzymatic actions of host and microbial alcohol dehydrogenase and aldehyde dehydrogenase, respectively, we also incubated *E. coli* in different concentrations of acetaldehyde and acetate. Additionally, we grew *E. coli* at pH 6 and pH 7 to mimic the normal pH range found in the left colon and rectum in humans.^27^ Although ethanol and acetaldehyde exhibited modest concentration-dependent inhibitory effects on *E. coli*, acetate robustly inhibited *E. coli* growth, leading to a prolonged lag phase and reduced stationary phase OD at higher concentrations. These inhibitory effects were most evident at pH 6 (Figure 4a–c) and less pronounced at pH 7 (Supplemental Figure S5a–c). Furthermore, the combination of these metabolites inhibited *E. coli* growth in a synergistic manner (Figure 4d).

### Neither stool nor rectal swab correlate with disease severity

Prior studies have shown that cirrhosis disease severity based on CPS or MELD score may correlate with taxonomic changes in the gut microbiome, where higher CPS and MELD may lead to greater dysbiosis.^8,9^ We utilized linear taxonomic modeling in our cirrhosis cohort to determine correlations between taxonomic changes and MELD. We did not identify any significant taxonomic changes associated with MELD (Table 2) in our cirrhosis cohort in either stool or rectal swab.

**Table 2.** Linear models of taxonomic changes with MELD and antibiotic use

Taxa changes in patients with higher MELD scores (rectal swab)			
Taxa	Estimate	*p*-value	FDR
*Veillonella parvula*	0.189	0.079	0.910
*Bifidobacterium longum*	−0.146	0.073	0.910
Taxa changes in patients with higher MELD scores (stool)			
*Clostridium bolteae*	−0.173	0.014	0.163
*Flavonifractor plautii*	−0.150	0.014	0.163
Taxa changes in patients given antibiotics (rectal swab)			
*Faecalibacterium prausnitzii*	−2.2	0.012	0.093
*Lachnoclostridium bolteae*	−1.9	0.0099	0.093
*Porphyromonas asaccharolytica*	−2.8	0.011	0.093
*Eubacterium rectale*	−1.8	0.029	0.17
Taxa changes in patients given antibiotics (stool)			
*Faecalibacterium prausnitzii*	−3	0.00048	0.0055
*Eubacterium rectale*	−3.3	0.00048	0.0055

### Antibiotic use modestly alters the gut microbiota composition in stool but not rectal swab

Since antibiotic use can affect the gut microbiota composition, we sought to determine whether it had an effect in our study cohort. Linear modeling of taxonomic changes showed that while there was no difference in rectal swab, antibiotic use led to modest alterations in the gut microbiota composition in stool with decreased abundances of two bacterial taxa, *Faecalibacterium prausnitizii* and *Eubacteria rectale* (FDR < 0.01, Table 2), emphasizing that stool and rectal swab may capture distinct niches within the gastrointestinal tract under varying degrees of influences from the host and/or environment. Rifaximin was the most commonly used antibiotic, with close to half of the study cohort on it for the treatment of hepatic encephalopathy (Table 1). We thus determined whether rifaximin alone had an effect on the gut microbiota composition. We did not identify significant taxonomic changes due to rifaximin either in stool or rectal swab, consistent with prior study showing that rifaximin use for hepatic encephalopathy in cirrhosis, while improving cognition and altering serum metabolites as well as bacterial network linkages, led to minimal changes in the composition of the gut microbiota.^28^

## Discussion

Although most gut microbiome studies utilize stool sampling due to ease of collection, it does not take into account the spatial distribution of the microbiota – an increasingly important consideration due to the effect of the environment on the composition and function of the microbiota. Longitudinal differences between the small bowel and colonic microbiota are an example of spatially defined environmental effects on the microbiota. In a similar fashion, there are also differences radially between the luminal and mucosa-associated microbiota.^13^ In this study, we sought to examine the luminal and mucosally adherent colonic microbiota in patients with cirrhosis using stool and rectal swabs, respectively. To our knowledge, this is the first study to characterize the gut microbiota in cirrhosis of different etiologies using rectal swab with taxonomic and functional analyses via shotgun metagenomic sequencing. We found important differences between the mucosal microbiome in rectal swab and the fecal microbiome in stool that may reflect intrinsic physiological differences between these two niches within the colon. The mucosal microbiome is enriched in two asaccharolytic bacterial taxa as well as genes/pathways involved in protein synthesis and cellular proliferation but decreased in genes/pathways related to carbohydrate metabolism.

Unlike the small bowel, where daily food intake provides a cyclical influx of nutrients to feed the host and a relatively smaller number of microorganisms, the colon is generally considered to be resource-limited with fierce competition among the dense microbial community for undigested and/or unabsorbed food. While dietary complex carbohydrates such as fiber may provide a carbon source for the colonic microbiota via fermentation and cross-feeding,^29^ nitrogen sources such as amino acids and peptides are generally scarce.^30^ In our study, we found that the relative abundances of two asaccharolytic (i.e. non-carbohydrate metabolizing) bacterial taxa, *Finegoldia magna* and *Porphorymonas asaccharolytica*, are increased in the rectal swabs relative to stool. Along with decreases in carbohydrate metabolic genes/pathways, there is an enrichment in genes/pathways related to protein synthesis and cellular proliferation. These findings suggest that the colonic mucosal microenvironment may be carbohydrate-limiting and/or nitrogen-abundant relative to the lumen. The sources of nitrogen likely include the rich glycoproteins in the mucus layer and/or shedding and turnover of the colonic epithelium.^31^ In agreement with these findings, prior work by our group as well as others have shown increased relative abundances of asaccharolytic bacteria including *Finegoldia, Porphorymonas, Anaerococcus, and Peptoniphilus* in the mucosal microbiome compared to the fecal microbiome.^13,18,32,33^

*Finegoldia magna* and *Porphorymonas asaccharolytica*, in addition to being asaccharolytic, are both obligate anaerobes as well as known opportunistic human pathogens. *F. magna* is a gram-positive anaerobic cocci (GPAC) that normally colonizes the skin and mucosal surfaces of the oral, respiratory, gastrointestinal, and female genitourinary tracts but can become opportunistic pathogens in immunocompromised hosts along with other GPACs, such as aforementioned *Anaerococcus* and *Peptoniphilus*.^34^ *Porphyromonas asaccharolytica*, on the other hand, is a gram-negative bacilli commonly found in the naso-oropharyngeal, gastrointestinal, and genitourinary tracts and has been found in brain abscesses, sinusitis, osteomyelitis, bacteremia, periodontal, pleuropulmonary, genitourinary, and soft tissue infections, as well as one reported case of liver abscess.^35^ Despite being obligate anaerobes, *F. magna* and *P. asaccharolytica* displayed higher relative abundances in the microaerobic mucosal environment, the spatial proximity of which may increase the likelihood for translocation relative to the lumen. In the setting of cirrhosis, translocation risk is further heightened by increased intestinal permeability and decreased immune defenses.^6^ Additionally, a previous study has shown that the mucosal, but not fecal, microbiome differ between those with and without hepatic encephalopathy.^36^ These findings emphasize the importance in studying the mucosal microbiome in predicting clinical outcome in cirrhosis.

Although no features in the fecal microbiome distinguished etiologies of cirrhosis, the mucosal microbiome revealed a taxonomic signature of alcohol-related cirrhosis with decreased abundances of *E. coli* and Enterobacteriaceae. Prior studies have examined whether the fecal microbiome differs according to the etiology of cirrhosis. Some studies have shown taxonomic signatures for specific cirrhosis etiologies, yet other studies have failed to demonstrate a difference.^8,9,24^ In our study, we did not detect a fecal microbial signature according to cirrhosis etiology. However, we found a mucosal microbial signature for alcohol-related cirrhosis with significantly lower relative abundances of *E. coli* and Enterobacteriaceae. One potential explanation for this finding may be the growth inhibitory effects of ethanol and its oxidative metabolites, acetaldehyde and acetate, on *E. coli* and Enterobacteriaceae. After ingestion, ethanol is mostly absorbed in the stomach and proximal small intestine, with minimal amount passing into the distal small intestine and colon.^37^ In addition to undergoing oxidation in the liver, ethanol can circulate throughout the body and diffuse into the colon at concentrations equivalent to that in the blood.^25,26^ Colonic epithelium and microbiota both possess alcohol dehydrogenase and aldehyde dehydrogenase activities that are able to oxidize ethanol into acetaldehyde and acetate.^38,39^ A previous study has shown that mucosal alcohol dehydrogenase activity may even be greater in the rectum than the rest of the colon.^40^ With acute or chronic alcohol consumption, microbes in the colon are exposed to ethanol likely not via direct longitudinal passage from the proximal gastrointestinal tract, but via diffusion across the intestinal epithelium from circulation and onto the mucosal surface, leading to greater ethanol-related microbial changes at the mucosal interface than the lumen. Using *in vitro* bacterial culture studies, we showed that at physiologic concentrations in the colonic environment after alcohol consumption, ethanol and acetaldehyde exhibited mild inhibitory effects on the growth of *E. coli*, while acetate exhibited a strong concentration-dependent inhibitory effect on *E. coli* growth most evident at pH of 6 and less pronounced at pH 7. It is indeed plausible that ethanol oxidation at the mucosal surface into acetaldehyde and acetate likely leads to a mild metabolic acidosis, enhancing their inhibitory effects on *E. coli*. Alcohols and aldehydes are widely used as antiseptics and disinfectants due to various antimicrobial mechanisms such as membrane damage, protein denaturation, cross-linking of macromolecules, and so on.^41^ The growth-inhibitory effects of ethanol and acetaldehyde on *E. coli* are thus perhaps not surprising, although limited studies have examined their antimicrobial activities at concentrations as low as those circulating in the body after alcohol consumption (for comparison, a blood alcohol content of 0.4, above which the likelihood of death ensues, is more than 100-fold less than 70% ethanol commonly used for disinfection). The mechanism(s) by which acetate inhibits *E. coli* and other Enterobacteriaceae are less well understood. Potential mechanisms posited in the literature include the uncoupling effect of acetic acid with resultant intracellular acidification and anion imbalance, methionine depletion and accumulation of toxic homocysteine, as well as perturbation of acetate metabolism due to excess acetate inflow and feedback inhibition.^42–44^

Among the various etiologies of cirrhosis, alcohol-related cirrhosis remains a major cause of morbidity and mortality worldwide. Most microbiome studies have not found a consistent dysbiosis signature unique to alcohol-related cirrhosis aside from the changes associated with chronic liver diseases and cirrhosis.^8,9,24,45,46^ Enterobacteriaceae, which belong to the Proteobacteria phylum and include *E. coli* and several other common human pathogens, have been found to be increased in the fecal microbiome of many disease states associated with inflammation, including cirrhosis. In our study, Enterobacteriaceae encompassed 10–20% of the bacteria in rectal swabs as well as in stool, whereas they generally make up <5% of the gut microbiome in health, suggesting that our cirrhotic cohort is indeed characterized by dysbiosis. Yet in rectal swabs, we found the relative abundances of *E. coli* and Enterobacteriaceae to be decreased in EtOH cirrhosis compared to non-EtOH cirrhosis, likely due to the inhibitory effect of ethanol oxidative metabolites near the mucosa. A recent study also found increased abundance of *Enterococcus faecalis* in the fecal samples of patients with alcoholic hepatitis,^47^ which was not seen in our study, although none of our patients was diagnosed with alcoholic hepatitis at the time of sample collection.

In the rectal swabs of patients with alcohol-related cirrhosis, we found a positive association between the abundances of *E. coli*/Enterobacteriaceae and the use of lactulose and/or Caucasian race. While lactulose can promote the growth of organisms such as *Lactobacillus* and *Bifidobacteria*, it has not been shown to promote *E. coli* and/or Enterobacteriaceae growth. One potential explanation for the higher abundances of *E. coli* and Enterobacteriaceae in patients on lactulose may be the osmotic diarrheal effects of lactulose within the colon that dilute the effects of ethanol and its oxidative metabolites on *E. coli* and Enterobacteriaceae near the colonic mucosa. Prior study has shown that even transient osmotic diarrhea induced by laxatives such as polyethylene glycol not only alters the gut microbiota long term but also disrupts the mucus barrier.^15^ In terms of the association between Caucasian ethnicity and *E. coli* abundance, prior studies have demonstrated that ethnic differences in gut microbiota composition are primarily due to geographic and cultural dietary preferences. All of our patients are from the Northeastern United States and thus likely consuming similar American diets. However, given the higher prevalence of lactase-persistence in people of Scandinavian descent^48^ and the biochemical and structural similarity between lactose and lactulose,^49^ Caucasian subjects are more likely to tolerate lactose and remain on lactulose, whereas non-Caucasian subjects may have higher prevalence of intolerance to lactose and lactulose. Indeed, no non-Caucasian subjects in the alcohol-related cirrhosis group were on lactulose despite some having documented hepatic encephalopathy.

There are limitations to our study. It was conducted at a single medical center in the Northeastern US with a limited sample size. Whether our findings can be generalized to the cirrhosis population at large in the US and worldwide will require further studies with larger sample sizes and a more heterogeneous study population. Additionally, our cirrhosis cohort consisted mostly of Child-Pugh Class B (CPS range 6–11, mean 7.67), with MELD score range 7–26 (mean 14.74). Thus, it is uncertain whether the findings can be extended to more severe liver diseases with higher CPS and MELD scores. We were unable to detect an association between dysbiosis and severity of liver disease potentially due to this limitation. Furthermore, although we hypothesized that ethanol and its metabolites are likely exerting greater effects at the mucosal surface due to diffusion gradient, we do not have correlative data between plasma, mucosal, and luminal levels of ethanol and its metabolites.

In conclusion, we have shown that the mucosal microbiome is taxonomically and functionally distinct from the luminal microbiome in cirrhosis and can be readily characterized via rectal swab. The finding of a taxonomic signature unique to alcohol-related cirrhosis in rectal swab, but not stool, reflects the unique environment inhabited by the mucosally associated microbiome. These findings suggest that mucosally-adherent microbiota provides important insight into the physiological interactions between the host and microbes and can be utilized for microbiome-based diagnostic and prognostic purposes in alcohol-related liver disease and cirrhosis.

## Patients and methods

### Patient population and inclusion/exclusion criteria

The samples used in this study are from the baseline rectal swab and stool samples of patients enrolled in the Acute-on-Chronic Liver Failure with Gut Microbiota-Targeted Assessment and Treatment (ACCLIMATE) study. ACCLIMATE is an ongoing longitudinal cohort study conducted at the Hospital of the University of Pennsylvania. The objective of ACCLIMATE is to determine whether the gut microbiome differs between patients with/without and before/after the development of acute-on-chronic liver failure (ACLF), thereby identify potential biomarkers and microbiota signature that may predict and prognosticate ACLF. Inclusion criteria include male or female age 18–80, confirmed diagnosis of cirrhosis based on imaging or biopsy along with appropriate history and clinical findings, Child-Turcotte-Pugh score 5–15, and subject or healthcare proxy capable of giving informed consent. Exclusion criteria include unclear diagnosis of cirrhosis, pregnant or lactating females, and unwillingness to provide informed consent. Patient clinical information are collected by reviewing their medical charts at the time of enrollment. This includes age, gender, ethnicity, cirrhosis etiology and method of diagnosis, cirrhosis history and complications (e.g. ascites, spontaneous bacterial peritonitis, hepatic encephalopathy, varices, hepatocellular carcinoma, transjugular intrahepatic portosystemic shunt, liver transplant listing, etc), comorbid medical conditions, medications, laboratory, pathology, and radiology evaluations. All research has been conducted in an ethical and responsible manner, and is in full compliance with all relevant codes of experimentation and legislation with the formal approval of the Institutional Review Board of the University of Pennsylvania (IRB protocol #827492). All mandatory laboratory health and safety procedures have been complied with in the course of conducting any experimental work. All participants provided written consent to the inclusion of material pertaining to themselves and acknowledge that they cannot be identified via the paper, and all data have been fully anonymized.

### Rectal swab and stool collection, processing, and storage

Rectal swab is performed by the study investigator or the participant’s primary hepatologist during clinic visit after informed consent is obtained. A sterile swab (Copan Diagnostics Nylon-Flocked Dry Swabs in Tubes, Catalog #23-600-964, Fisher Scientific) is inserted 3 cm into the rectum, turned 360°, removed, placed into a sterile tube, and stored frozen at −80°C within 30 minutes of collection. A control swab is waved in the air, placed into a sterile tube, and stored frozen at −80°C. Stool is collected after the rectal swab prior to leaving the clinic if possible. If a participant is unable to provide stool prior to leaving the clinic, he or she is provided a stool collection kit to take home and instructed to return it within 1 week. Stool collection and aliquoting instructions are provided and verbally explained to all participants in advance. The participant is also asked to complete a form identifying the stool sample from the standard Bristol Stool Chart. Stool samples must be collected no more than 24 hours before given to UPS for shipping. Upon receipt of the package containing the stool specimens, processing occurs in dedicated biohood in the laboratory. A tube containing ethanol is stored at −80**°**C. The excess stool is weighed and aliquoted as follows and stored at −80**°**C: 4 spoon-top tubes (Sarstedt) – 3 dry and 1 containing RNALater®, 1 coring tube (Globe Scientific) to obtain frozen stool cores for use with CryoXtract 350.

### Shotgun metagenomic sequencing and statistical analysis

Shotgun metagenomic analysis of rectal swab and stool samples are performed by the Sequencing and Analytical Center of the PennCHOP Microbiome Program to determine all of the genetic material available within the given samples. To do this, DNA are purified from each sample using the MoBio PowerSoil kit. Libraries for DNA sequencing are prepared using the TruSeq method, and sequences are acquired using the Illumina HiSeq method. Shotgun metagenomic data are analyzed using Sunbeam, a user-extendable bioinformatics pipeline that we developed for this purpose.^50^ Quality control steps are performed by the default workflows in Sunbeam, which are optimized to remove host-derived sequences and reads of low sequence complexity. The abundance of bacteria are estimated using Kraken.^51^ Reads are mapped to the KEGG database^52^ using Diamond^53^ to estimate the abundance of bacterial gene orthologs. Sample similarity are assessed by Bray-Curtis and Jaccard distances, and community-level differences between sample groups are assessed using the PERMANOVA test. Linear models were used to detect differences in logit-transformed gene and taxon abundance between sample groups. *P*-values from multiple testing procedures were corrected to control for a false discovery rate of 0.05. Continuous variable statistics are computed by Student’s t-tests. Categorical variable statistics are computed by Fisher’s exact test.

### In vitro bacterial culture experiments

*Escherichia coli* type strain ATCC 11775 (American Type Culture Collection, NCTC 9001) was incubated in lysogeny broth (LB) at 37°C under aerobic condition to grow to saturation overnight. The next morning, *E. coli* was inoculated at 1:100 dilution into fresh LB media in 96-well plates containing different concentrations of ethanol, acetaldehyde, and/or acetate titrated to pH 6 and 7 to reflect physiological pH range in the left colon and rectum. Ethanol concentrations (12.5 mM, 25 mM, 50 mM, 100 mM) were calculated to reflect different blood alcohol concentrations (BAC) diffusing into the colon, including low (12.5 mM = BAC 0.058), moderate (25 mM = BAC 0.115), high (50 mM = BAC 0.23), and dangerously high (100 mM = BAC 0.46) levels. Acetaldehyde concentrations in the colon (31.25 uM, 62.5 uM, 125 uM, 250 uM) were determined based on literature.^54^ Acetate concentrations (12.5 mM, 25 mM, 50 mM, 100 mM) were determined based on 1:1 ethanol oxidation as well as reported acetate levels in the colon.^55^ The plates were placed into automated plate reader and cultured for 24-hours under aerobic condition to reflect the environment at the mucosal surface in the rectum. Optical density at 600 nm was taken every hour and plotted to generate bacterial growth curves.

## Supplementary Material

Supplemental Material

Supplemental Material

Supplemental Material

## Data Availability

The data that support the findings of this study are available at https://www.ncbi.nlm.nih.gov/bioproject/PRJNA766067
