# Supplementary figures and images for: The Mucosally-Adherent Rectal Microbiota Contains Features Unique to Alcohol-Related Cirrhosis
Source: Gut Microbes. 2021 Nov 7;13(1):1987781. doi: 10.1080/19490976.2021.1987781 (PMC8583005; doi:10.1080/19490976.2021.1987781)

# Supplemental Figure 1

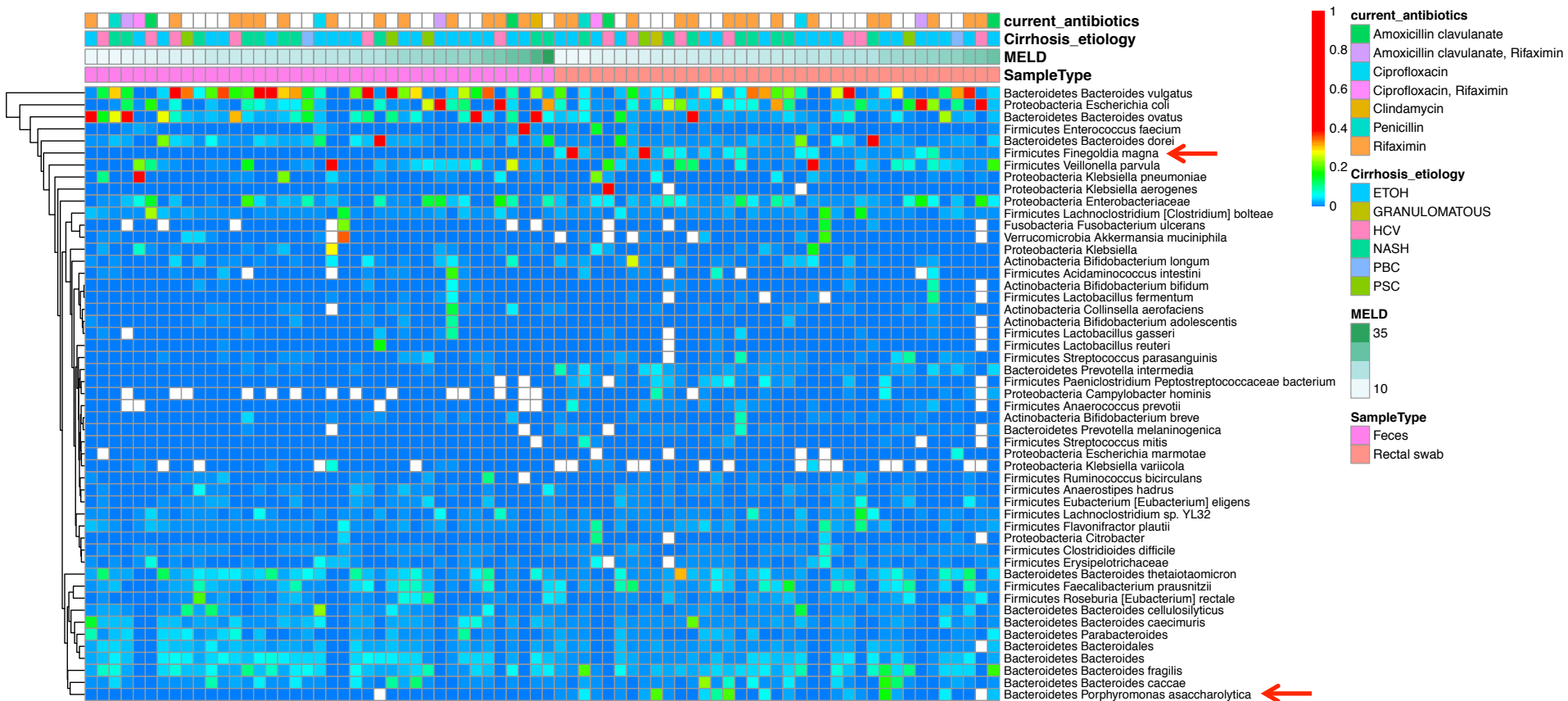

**A**

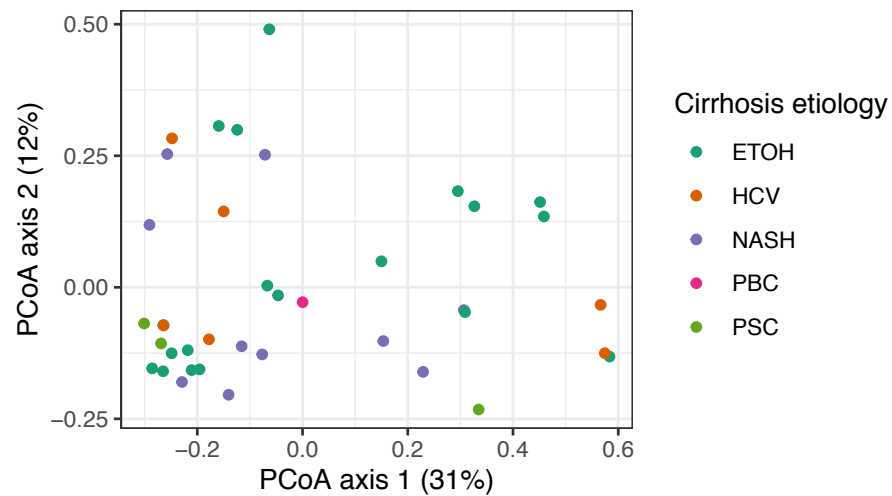

**B**

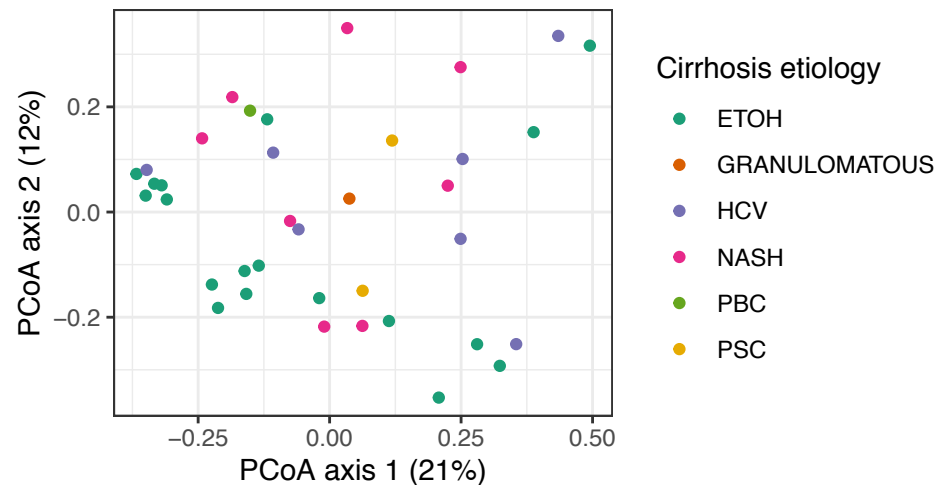

Supplemental Figure 3

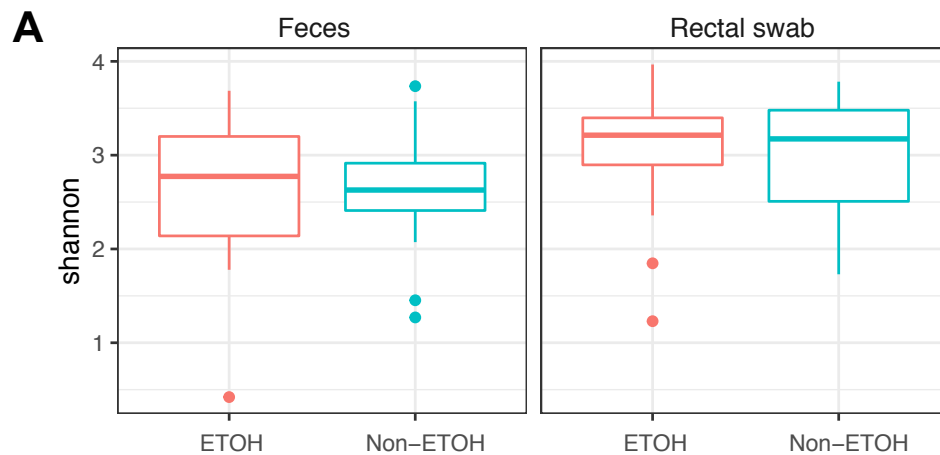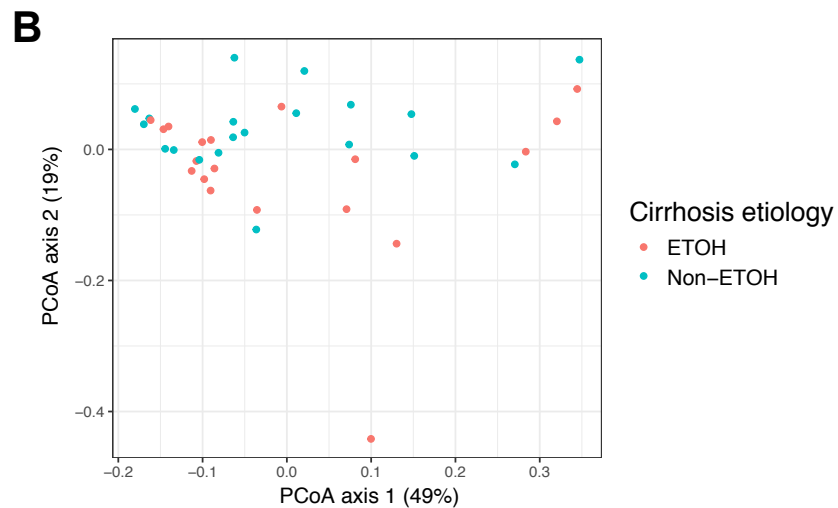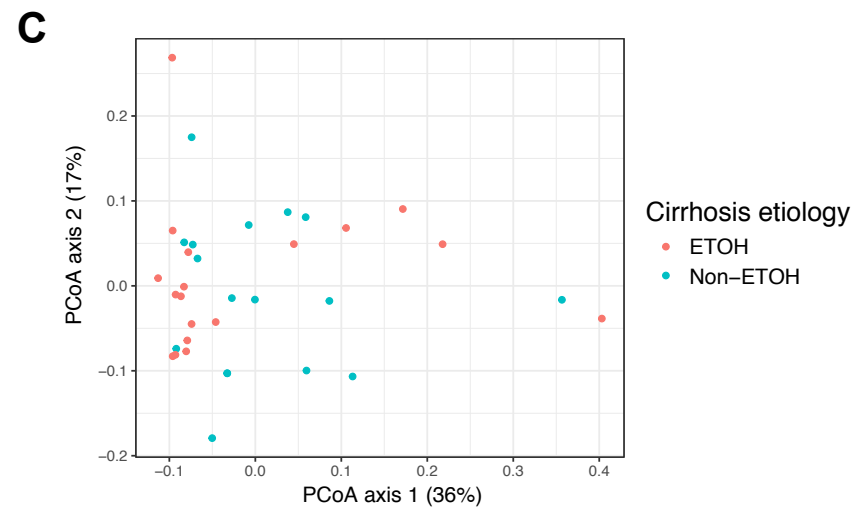

**A**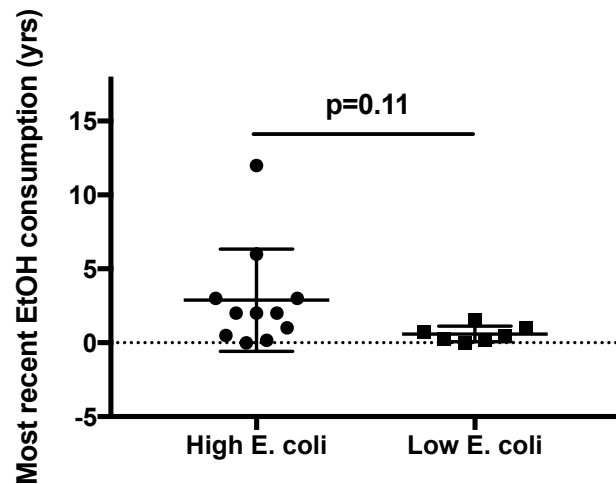**B**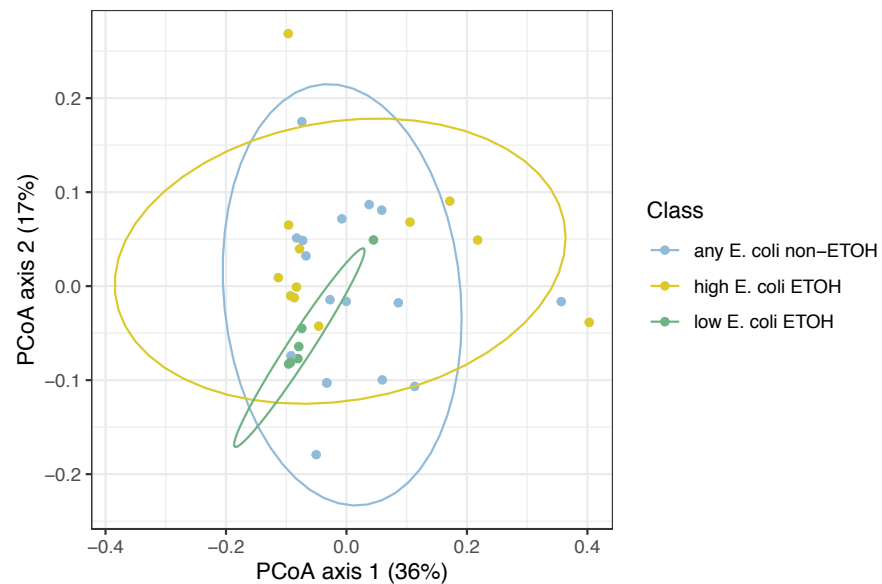

Supplemental Figure 5

**A**

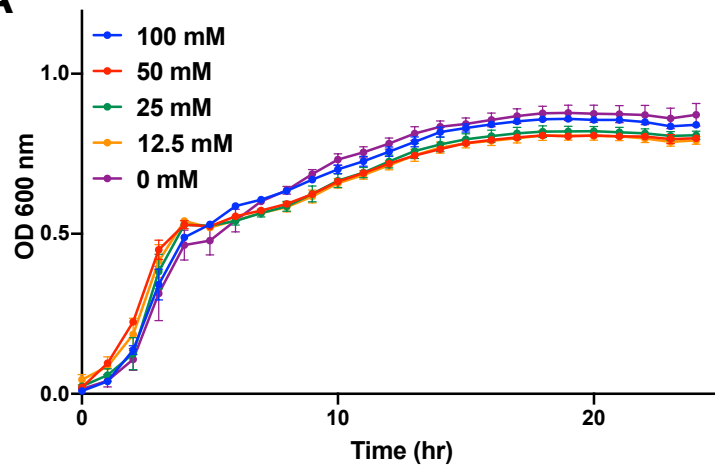

**B**

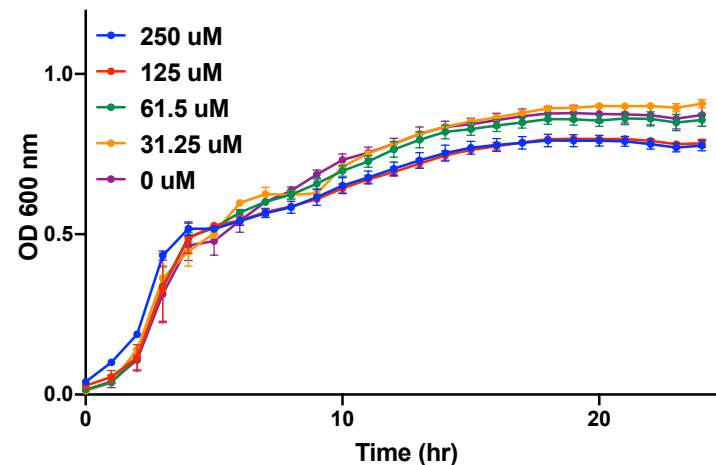

**C**

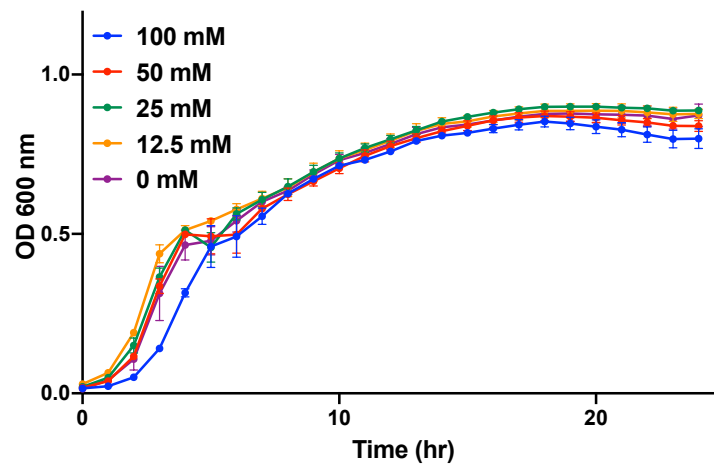

Supplement: Supplemental Material [file KGMI_A_1987781_SM5927.pdf]
