# Supplementary material for: The Mucosally-Adherent Rectal Microbiota Contains Features Unique to Alcohol-Related Cirrhosis
Source: Gut Microbes. 2021 Nov 7;13(1):1987781. doi: 10.1080/19490976.2021.1987781 (PMC8583005; doi:10.1080/19490976.2021.1987781)
Supplement: Supplemental Material [file KGMI_A_1987781_SM5503.pdf]

Supplemental Figure 1. Heatmap of taxonomic assignments in stool and rectal swabs. Samples are clustered by sample type and MELD score. Etiology of cirrhosis and antibiotic use are coded in bars above the heatmap with the legends indicated to the right. Each column represents one sample and each row represents one taxon. The relative abundance is color coded with the index shown to the right of the heatmap. Red arrows indicate taxa that differ significantly in relative abundance between fecal and rectal swab samples.

Supplemental Figure 2. Beta diversity of taxonomy in fecal and rectal swab samples by cirrhosis etiology. (A) Principal Coordinates Analysis via Bray-Curtis distances of stool by cirrhosis etiology (p=0.902). (B) Principal Coordinates Analysis via Bray-Curtis distances of rectal swab by cirrhosis etiology (p=0.992).

Supplemental Figure 3. Alpha diversity between EtOH and non-EtOH cirrhosis and Bray-Curtis distances of gene abundance. (A) Alpha-diversity (Shannon) between EtOH and non-EtOH cirrhosis in stool (p=0.97) and rectal swab (p=0.64). (B) PCoA (Bray-Curtis) of KEGG ortholog terms between EtOH and non-EtOH cirrhosis in feces (R2=0.034, Permanova=0.22). (C) PCoA (Bray-Curtis) of KEGG ortholog terms between EtOH and non-EtOH cirrhosis in rectal swab (R2=0.013, Permanova=0.92).

Supplemental Figure 4. Most recent alcohol consumption and β-diversity of KEGG term abundances in high *E. coli*/Enterobacteriaceae versus low *E. coli*/Enterobacteriaceae groups. (A) Most recent alcohol consumption from time of sample collection in high and low *E. coli*/Enterobacteriaceae groups in rectal swabs of EtOH cirrhosis (p=0.11). (B) PCoA (Bray-Curtis) of KEGG ortholog terms in rectal swabs of high *E. coli*/Enterobacteriaceae group, low *E. coli*/Enterobacteriaceae group, and non-EtOH cirrhosis (R2=0.073, FDR=0.22).

Supplemental Figure 5. *E. coli* growth *in vitro* under physiological concentrations of ethanol after alcohol consumption and its oxidative metabolites at pH 7. Growth curve in LB (aerobic, 37°C) with (A) ethanol, (B) acetaldehyde, and (C) acetate. Data expressed as mean±SEM. N=3 replicates per condition.
