# Supplementary material for: The Mucosally-Adherent Rectal Microbiota Contains Features Unique to Alcohol-Related Cirrhosis
Source: Gut Microbes. 2021 Nov 7;13(1):1987781. doi: 10.1080/19490976.2021.1987781 (PMC8583005; doi:10.1080/19490976.2021.1987781)
Supplement: Supplemental Material [file KGMI_A_1987781_SM9286.docx]

| **GeneID** | **Rectal swab to stool** | **Estimate** | **FDR** | **Definition** | **Function** |
| --- | --- | --- | --- | --- | --- |
| K03205 | increased | 0.89 | 1.60E-02 | type IV secretion system protein VirD4 | virulence |
| K06147 | increased | 0.6 | 3.50E-05 | ATP-binding cassette, subfamily B, bacterial | transmembrane transport |
| K03763 | increased | 0.48 | 7.50E-03 | DNA polymerase III | replication |
| K02035 | increased | 0.41 | 9.20E-03 | peptide/nickel transport system substrate-binding protein | cell transport |
| K02358 | increased | 0.37 | 2.60E-06 | elongation factor Tu | translation |
| K00789 | increased | 0.32 | 3.50E-05 | S-adenosylmethionine synthetase | transcription, cell proliferation |
| K03737 | increased | 0.3 | 4.50E-04 | pyruvate- ferredoxin/flavodoxin oxidoreductase | pyruvate and acetyl-CoA interconversion |
| K01881 | increased | 0.28 | 3.50E-05 | prolyl-tRNA synthetase | tRNA synthetase |
| K02355 | increased | 0.27 | 1.90E-04 | elongation factor G | translation |
| K03798 | increased | 0.27 | 8.10E-04 | cell division protease FtsH | cell division |
| K00088 | increased | 0.26 | 3.40E-04 | IMP dehydrogenase | purine biosynthesis |
| K05366 | increased | 0.26 | 4.00E-04 | penicillin-binding protein 1A | cell wall formation |
| K03076 | increased | 0.25 | 3.50E-05 | preprotein translocase subunit SecY | protein transport |
| K00525 | increased | 0.24 | 5.10E-04 | ribonucleoside- diphosphate reductase alpha chain | DNA synthesis |
| K01868 | increased | 0.24 | 4.80E-04 | threonyl-tRNA synthetase | tRNA synthetase |
| K03495 | increased | 0.24 | 3.50E-05 | tRNA uridine 5-carboxymethylaminomethyl modification enzyme | translation |
| K03702 | increased | 0.24 | 4.70E-04 | excinuclease ABC subunit B | DNA repair |
| K01874 | increased | 0.23 | 3.30E-04 | methionyl-tRNA synthetase | tRNA synthetase |
| K01952 | increased | 0.23 | 1.90E-04 | phosphoribosylformylglycinamidine synthase | purine metabolism |
| K02469 | increased | 0.23 | 2.00E-03 | DNA gyrase subunit A | replication/transcription |
| K03070 | increased | 0.23 | 1.10E-03 | preprotein translocase subunit SecA | protein transport |
| K03596 | increased | 0.23 | 1.10E-03 | GTP-binding protein LepA | cell signaling membrane |
| K03695 | increased | 0.23 | 7.10E-03 | ATP-dependent Clp protease ATP-binding subunit ClpB | protein degradation |
| K04043 | increased | 0.22 | 2.30E-03 | molecular chaperone DnaK | DNA replication, stress response |
| K00962 | increased | 0.21 | 4.80E-04 | polyribonucleotide nucleotidyltransferase | mRNA degradation |
| K01870 | increased | 0.21 | 9.10E-04 | isoleucyl-tRNA synthetase | tRNA synthetase |
| K02470 | increased | 0.21 | 2.90E-03 | DNA gyrase subunit B | replication/transcription |
| K03043 | increased | 0.21 | 1.50E-04 | DNA-directed RNA polymerase subunit beta | transcription |
| K04077 | increased | 0.21 | 2.00E-03 | chaperonin GroEL | protein folding |
| K01869 | increased | 0.2 | 4.50E-03 | leucyl-tRNA synthetase | tRNA synthetase |
| K01883 | increased | 0.2 | 1.80E-03 | cysteinyl-tRNA synthetase | tRNA synthetase |
| K03168 | increased | 0.2 | 6.10E-03 | DNA topoisomerase I | replication/transcription |
| K06207 | increased | 0.2 | 4.50E-04 | GTP-binding protein | cell signaling |
| K00790 | increased | 0.19 | 3.50E-03 | UDP-N- acetylglucosamine 1-carboxyvinyltransferase | cell wall formation |
| K01752 | increased | 0.19 | 4.80E-04 | L-serine dehydratase | gluconeogenesis |
| K04567 | increased | 0.19 | 5.40E-05 | lysyl-tRNA synthetase | tRNA synthetase |
| K01873 | increased | 0.18 | 2.30E-03 | valyl-tRNA synthetase | tRNA synthetase |
| K03046 | increased | 0.18 | 9.60E-04 | DNA-directed RNA polymerase subunit beta | transcription |
| K01534 | increased | 0.17 | 7.10E-03 | Zn2+/Cd2+-exporting ATPase | cell membrane transport |
| K01689 | increased | 0.17 | 1.60E-02 | enolase | glycolysis |
| K01872 | increased | 0.16 | 2.00E-03 | alanyl-tRNA synthetase | tRNA synthetase |
| K02519 | increased | 0.16 | 4.50E-03 | translation initiation factor IF-2 | translation |
| K01756 | increased | 0.15 | 2.20E-02 | adenylosuccinate lyase | purine biosynthesis |
| K01887 | increased | 0.15 | 1.70E-02 | arginyl-tRNA synthetase | tRNA synthetase |
| K02335 | increased | 0.15 | 3.70E-03 | DNA polymerase I | replication |
| K03544 | increased | 0.15 | 2.70E-02 | ATP-dependent Clp protease ATP-binding subunit ClpX | protein degradation |
| K03703 | increased | 0.15 | 1.40E-02 | excinuclease ABC subunit C | DNA repair |
| K01937 | increased | 0.14 | 7.40E-03 | CTP synthase | pyrimidine biosynthesis |
| K01972 | increased | 0.14 | 1.90E-03 | DNA ligase | replication |
| K01939 | increased | 0.13 | 1.40E-02 | adenylosuccinate synthase | purine biosynthesis |
| K03466 | increased | 0.13 | 1.10E-02 | DNA segregation ATPase FtsK/SpoIIIE, S-DNA-T family | cell division |
| K12573 | increased | 0.12 | 7.40E-03 | ribonuclease R | RNA degradation |
| K08303 | increased | 0.1 | 2.90E-02 | putative protease | protein degradation |
| K00873 | increased | 0.09 | 2.00E-02 | pyruvate kinase | glycolysis |
| K02529 | decreased | -0.26 | 1.80E-03 | LacI family transcriptional regulator | lactose metabolism |
| K06889 | decreased | -0.41 | 1.10E-03 | uncharacterized protein | N/A |
| K07407 | decreased | -0.53 | 2.00E-02 | alpha-galactosidase | carbohydrate metabolism |
| K01187 | decreased | -0.63 | 8.50E-04 | alpha-glucosidase | carbohydrate metabolism |

**Supplemental Table 1.** Linear models of logistic transformed abundances of genes with >0.1% abundance that differ between rectal swab and stool.
